# Long Non-coding RNA *MSTRG.24008.1* Regulates the Regeneration of the Sciatic Nerve via the miR-331-3p–NLRP3/MAL Axis

**DOI:** 10.3389/fcell.2021.641603

**Published:** 2021-06-04

**Authors:** Gang Yin, Ying Peng, Yaofa Lin, Peilin Wang, Zhuoxuan Li, Renyuan Wang, Haodong Lin

**Affiliations:** Department of Orthopedic Surgery, Shanghai General Hospital, Shanghai Jiao Tong University School of Medicine, Shanghai, China

**Keywords:** long non-coding RNA, peripheral nerve injury, neural regeneration, *MSTRG.24008.1*, miR-331-3p, NLRP3, MAL 3

## Abstract

Peripheral nerve injury (PNI) is a common clinical problem, which can cause severe disability and dramatically affect a patient’s quality of life. Neural regeneration after PNI is a complex biological process that involves a variety of signaling pathways and genes. Emerging studies demonstrated that long non-coding RNAs (lncRNAs) were abnormally expressed after PNI and played pivotal roles in peripheral nerve regeneration. Based on the rat sciatic nerve injury model, we found that the expression levels of several lncRNAs were increased significantly in the sciatic nerve after injury. Software prediction prompted us to focus on one up-regulated lncRNA, *MSTRG.24008.1*. Dual-luciferase reporter assay, RNA pull-down assay and RNA interference approach verified that *MSTRG.24008.1* regulated neuroregeneration via the miR-331-3p/nucleotide-binding oligomerization domain-like pyrin domain containing 3 (NLRP3)/myelin and lymphocyte protein (MAL) axis *in vitro*. Subsequently, we performed gastrocnemius muscle gravity and sciatic functional index experiments to evaluate the recovery of injured sciatic nerves after *MSTRG.24008.1* siRNA interference *in vivo*. In conclusion, knockdown of *MSTRG.24008.1* promotes the regeneration of the sciatic nerve via the miR-331-3p/NLRP3/MAL axis, which may provide a new strategy to evaluate and repair injured peripheral nerves clinically.

## Introduction

Peripheral nerve injury (PNI) is a common clinical problem that is mostly caused by trauma and medical disorders, resulting in a financial burden to healthcare systems (Grinsell and Keating, 2014). With the intrinsic growth capacity of neurons and the permissive microenvironment mostly provided by Schwann cells, the peripheral nervous system (PNS) has a regenerative ability, which is different from the central nervous system (CNS) (Yao and Yu, 2019). However, because of the slow regeneration speed and other factors, such as the severity and site of the nerve injury, the regeneration distance, axonal excitability, and the inflammatory milieu, it takes a long time for axons to regenerate and reach the target organ. Before the target organ regains innervation, muscles might have atrophied irreversibly (Cobianchi et al., 2017; Weng et al., 2018). Therefore, further exploration of the biological process of neuroregeneration may provide new therapeutic targets. In addition, how to accelerate the speed of neuroregeneration remains an important issue that requires urgent study. Accompanied with the dedifferentiation of Schwann cells and activation of an immune response, Wallerian degeneration occurs after PNI in the distal segment of the lesion and breaks down both axons and myelin (Chen et al., 2015). The dedifferentiated Schwann cells proliferate and migrate to the lesion to clear myelin and axon debris, form Bungner bands, and excrete neurotrophic factors, and therefore provide a suitable microenvironment for neuroregeneration (Glenn and Talbot, 2013; Wu and Murashov, 2013). Importantly, a previous study showed that large numbers of long non-coding RNAs (lncRNAs) were involved in this biological process (Yao and Yu, 2019).

Non-coding RNAs, including circular RNAs (circRNAs) lncRNAs, and microRNAs, do not encode proteins, but can regulate many biological and pathological processes (Slack and Chinnaiyan, 2019). LncRNAs are the major class of ncRNAs that are longer than 200 nucleotides (Rinn and Chang, 2012). They regulate many biological processes on the epigenetic, transcriptional, and posttranscriptional levels (Liu et al., 2017). Recent studies demonstrated that a series of lncRNAs, which were verified to be very significant for neuroregeneration research, were differentially expressed after PNI, such as *BC089918*, *Uc.217*, *Silc1*, and *NON-MMUG014387* (Yu et al., 2013; Yao et al., 2015; Pan et al., 2017; Perry et al., 2018). Overexpression or knockdown of these lncRNAs could promote neurite outgrowth or Schwann cells proliferation significantly.

Nucleotide-binding oligomerization domain-like pyrin domain containing 3 (NLRP3) is one member of inflammasomes. Related research (Atianand et al., 2013) showed that, as an important component of innate immunity, the NLRP3 inflammasome played the major role in body’s immune response and disease development. NLRP3 activation can induce inflammatory necrosis of cells and aggravate local inflammatory responses and tissue destruction. Therefore, NLRP3 has become a new target for the treatment of various diseases (Pirzada et al., 2020). Myelin and lymphocyte protein (MAL) plays an important role in compacting myelin. MAL is a component of myelin and is expressed predominantly in oligodendrocytes in the CNS and in myelinating Schwann cells in the PNS (Schaeren-Wiemers et al., 1995).

In the present study, we identified a series of injury-related lncRNAs that were differentially expressed in the sciatic nerve after injury. Based on software prediction, we subsequently decided to focus on lncRNA *MSTRG.24008.1*, one of the lncRNAs that showed upregulated expression in the injured sciatic nerve. We revealed that NLRP3 and *MAL* were downstream targets of *MSTRG.24008.1*. Silencing of *MSTRG.24008.1* by small interfering RNAs resulted in downregulated expression of NLRP3 and MAL, and promoted Schwann cell proliferation and neuron repair. Thus, the results of this study identified a crucial lncRNA in PNI and neuroregeneration.

## Materials and Methods

### Animal Model Building

Adult male Wistar rats (weighing 240–260 g) were purchased from Slac Laboratory Animal Co. Ltd. and all the animal experiments were approved by the Research Ethics Committee of Shanghai General Hospital. Animal models of sciatic nerve injury were established according the reported methods (De et al., 1986; Savastano et al., 2014). The rats were anesthetized using the intraperitoneal injection of 1% sodium pentobarbital at 40 mg/kg. The sciatic nerve was crushed with the forceps at the force of 5 kg for 3 times (5 s each time, 10 s for interval) at 6–8 mm distal to the ischial tuberosity. The width of the nerve trunk crush area was about 3 mm. Marked with a 9–0 non-invasive suture, the sciatic nerve was replaced back to its original position. After 10 min, the rats were tested for the action potential latency, amplitude, and motor nerve conduction velocity (MCV). The injury model was judged to be constructed successfully if the MCV dropped to 10 m/s or lower. After anesthesia, the rats in the sham operation group were only operated to expose the left sciatic nerve, but whose nerves were not clamped. The control rats were marked in the same way as the model rats and sutured.

### Schwann Cell Culture, Plasmid Construction

Schwann cells were separated according to the previous report (Cobianchi et al., 2017). The Schwann cells were isolated from the site of sciatic nerve injury and 5 mm at the distal end at 48 h after sciatic nerve injury. Schwann cells were cultured under the condition of 37°C and 5% CO2 in Schwann cell specific medium. The *MSTRG.24008.1* cDNA was synthesized by GenScript Biotech (Nanjing, China). The *MSTRG.24008.1* small interfering RNA (siRNA), miR-331-3p agomir, miR-331-3p antagomirs, *NLRP3* siRNA, *MAL* siRNA, and their respective controls were synthesized by GenePharma Co. (Shanghai, China). Vectors expressing these nucleic acids were transfected into Schwann cells using Lipofectamine 2000 (Invitrogen, United States).

### Lentivirus Production and Transfections

Lentivirus production and transfections were performed according to the previous report (Elegheert et al., 2018). In brief, 24 h before lentiviral transfection, the Schwann cells were laid into 24 well plates with 1 × 10^5^/well. On the second day, the number of cells was about 2 × 10^5^/well. The original medium was replaced with 2 ml fresh medium containing 6 μg/ml Polybrene. Thereafter, the lentivirus solution was added to the cells according to different multiplicities of infection (MOIs), and mixed gently at 37°C. The cells were then incubated for about 12 h. The virus-containing culture medium was then aspirated, replaced with fresh culture medium, and the incubation was continued at 37°C for 48 h. Cells were collected 48 h after lentivirus infection and western blotting was used to determine the overexpression or downregulation efficiency.

After anesthetized, the rats were placed in prone position. After a small incision was formed in the intervertebral space between L3-L4 vertebrae, the sterile PE10 intrathecal catheter was inserted into the lumbar spine through the incision. Three days later, animal models of sciatic nerve injury were established. Lentivirus-NC or Lentivirus-MSTRG.24008.1 (4.0 × 108 TU/ml, 10 μl) was injected through PE 7 days after SNI model were established.

### High-Throughput RNA-Sequencing (RNA-seq) and Quantitative Real-Time Reverse Transcription PCR

Total RNAs were extracted from the sciatic nerve and all the ribosomal RNAs were removed. The RNAs were then broken into fragments and connected to sequencing adapters. PCR amplification was performed to construct a sequencing library, which was then subjected to sequencing on an Illumina HiSeq 4000 instrument (Illumina, San Diego, CA, United States) in the PE150 (150 bp of paired end sequence) sequencing mode.

For quantitative real-time reverse transcription PCR (qRT-PCR), total RNA was extracted from the sciatic nerve and cultured Schwann cells using the Trizol reagent (TransGen Biotech, Beijing, China) and reverse transcribed to cDNA using a FastKing RT Enzyme Mix (Tiangen, Beijing, China). Quantitative real-time PCR (qPCR) reactions were performed on ABI 7500 instrument (Applied Biosystems, Foster City, CA, United States). *GAPDH* (encoding glyceraldehyde-3-phosphate dehydrogenase; for *MSTRG.24008.1*, *NLRP3*, and *MAL*) and U6 snRNA (for miR-331-3p) were used as internal references. The primers were as follows: *MSTRG.24008.1* forward 5′-AGATCAAGCACACTCCCACA-3′, reverse 5′-CTA GCTTCCTACCACCCACC-3′; *NLRP3* forward 5′-GTAGGTG TGGAAGCAGGACT-3′, reverse 5′-CTTGCTGACTGAGGAC CTGA-3′; *MAL* forward 5′-TGGGGAGACTTCCTGGATCA-3′, reverse 5′-TCGAACATTGTGATGGTGGC-3′; *GAPDH* forward 5′-ACAGCAACAGGGTGGTGGAC-3′, reverse 5′-TTTGAGGG TGCAGCGAACTT-3′.

### Biotin-Labeled miRNA Pull-Down Assay

Biotin-coupled MSTRG.24008.1 and a negative control probe were designed and synthesized by RiboBio (Guangzhou, China). The sequence of the biotin-coupled MSTRG.24008.1 probe was TACCTGTGTGCTTGAGAATG. The biotin-coupled RNA complex was pulled down by incubating the streptavidin-coated magnetic beads with the cell lysates. The pull-down product was mixed with C-1 magnetic beads (Life Technologies) at 4°C for 3 h and then washed with wash buffer. The magnetic breads were incubated with proteinase K and lysis buffer to break the formaldehyde cross-links. Finally, the bound RNAs were extracted by qRT-PCR for the analysis.

### Dual-Luciferase Reporter Assay

The *MSTRG.24008.1* cDNA fragment and the *NLRP3* 3′-untranslated region containing the predicted potential rno-miR-331 binding site were ligated into the pYr-MirTarget luciferase reporter vector (YRBio) to generate pYr-MirTarget-MSTRG.24008.1 (MSTRG.24008.1^WT^) and pYr-MirTarget-NLRP3 (NLRP3^WT^) vectors (WT = wild-type). The mutant (MUT) rno-miR-331 binding sites were replaced as indicated to construct the pYr-MirTarget-MSTRG.24008.1-MUT (MSTRG.24008.1^MUT^) and pYr-MirTarget-NLRP3-MUT (NLRP3^MUT^) vectors. Schwann cells were cultured overnight at 37°C, 5% CO_2_ and saturated humidity. The cells were then co-transfected with 2 μg of the constructed plasmid vector and 2 μg of the rno-miR-331 agomir or agomir NC (negative control) using Lipofectamine^TM^ 2000 (Invitrogen, United States). At 24 h after transfection, the luciferase activity in the cells was evaluated using a Dual-Luciferase Assay Kit (Beyotime, Jiangsu, China).

### 3-(4,5-Dimethylthiazol-2-yl)-2,5-Diphenyltetrazolium Bromide (MTT) Cell Proliferation Assay

MTT (25 mg) was dissolved in 5 ml of MTT solvent. Then, 10 μl of this MTT solution was added to each well at the required detection time point, and incubated for 4 h in the cell incubator of Formazan dissolving solution (100 μl) was added to each well and mixed appropriately. Incubation was continued in the cell incubator at 37°C until the Formazan was completely dissolved, as observed under a light microscope. The absorbance was then measured at 570 nm using a spectrophotometer.

### 5-Ethynyl-20-deoxyuridine (Edu) Cell Proliferation Assay

The proliferation of Schwann cells was analyzed by Edu assay. The logarithmic growth phase cells were planted into 96-well plates with 1 × 10^5^ cells/ml, and then cultured for another 48 h. Cell proliferation was determined by Edu assay kit (Beyotime, Shanghai, China) according to the manufacturer’s instructions. The images of Schwann cells were detected by a fluorescence microscope (Leica, Germany) with Hoechst 33342 nucleus staining.

### Footprint Function and Scoring Analysis

Two weeks after surgery, the hind paws of the rats were immersed in a 1% bromophenol blue solution and the rats were induced to walk on paper strips (the strips covered a glass walking track with a length of 140 cm in and a width of 10 cm). Paw length (PL), toe opening distance (TS) and middle toe opening distance (IT) were measured from the recorded paw prints. The Sciatic Functional Index (SFI) was measured by the following formula according to previous literature (Pavic et al., 2008).

SFI=109.5⁢[(ETS-NTS)/NTS]-38.3⁢[(EPL-NPL)/NPL]+13.3⁢[(EIT-NIT)/NIT]-8.8

Note: ETS, EPL and EIT represent the PL, TS, and IT on the experimental side, respectively. Similarly, NTS, NPL, and NIT represent the PL, TS, and IT on the control side. For SFI, 0 indicates normal neurological function and 100 indicates severe injury.

### Gastrocnemius Wet Weight Measurement

Rats were sacrificed using an intraperitoneal injection of excessive 1% sodium pentobarbital at 0, 2, and 4 weeks after surgery. The experimental gastrocnemius muscle was completely removed, blood was washed off, and the muscle was weighed immediately on an electronic balance to calculate the gastrocnemius muscle gravity (GMG).

### Terminal Deoxynulceotidyl Transferase Nick-End-Labeling (TUNEL) Staining

A sciatic nerve specimen embedded in paraffin was deparaffinized using xylene for 5–10 min and then the xylene was replaced with fresh xylene and the specimen was deparaffinized for another 5–10 min. The sample was incubated in anhydrous ethanol for 5 min, 90% ethanol for 2 min, 70% ethanol for 2 min, and distilled water for 2 min. DNase-free proteinase K (20 μg/ml) was added dropwise at 37°C for 15–30 min, and then the sample was washed three times with phosphate-buffered saline (PBS). Then, 50 μl of TUNEL detection solution was added to the sample, which was incubated at 37°C in the dark for 60 min. After mounting using an anti-fluorescence quenching mounting solution, the sample was observed under a fluorescence microscope. The excitation wavelength range used was 450–500 nm, and the emission wavelength range was 515–565 nm.

### Hematoxylin and Eosin Staining and Fluorescence *in situ* Hybridization

Hematoxylin and Eosin (H&E) staining and Fluorescence *in situ* hybridization (FISH) were performed according to our previously described procedures (Liao et al., 2019). The sham and SNI sciatic nerve were observed by routine H&E staining. All nerve tissues were fixed in 10% formaldehyde for 24 h, and then were dehydrated, permeated, wax-dipped, and embedded in paraffin. The tissues were subsequently cut into 3μm sections and then stained with hematoxylin and eosin. S100B, MAP2, and *MSTRG.24008.1* probes were designed and synthesized by RiboBio (Guangzhou, China). The probe signals were detected with a FISH Kit (RiboBio, Guangzhou, China) according to the manufacturer’s instructions. Briefly, SCs were fixed in 4% formalin for 15 min. After rehybridization in PBS, the cells were hybridized at 37°C for 30 min in hybridization solution. Then, cell nuclei underwent counterstaining by utilizing DAPI (Beyotime, China). Images were captured using a fluorescence microscope (Leica, Germany).

### Electron Microscopy

After the sciatic nerve specimen was fixed with glutaraldehyde for 48 h, it was dehydrated in 50% acetone solution (once for 10 min), then in 70% acetone solution (once for 10 min), and then in 90% acetone solution (twice for 10 min each). The sample was then incubated in pure acetone-propylene oxide 812 embedding agent at room temperature for 2 h (the sample was placed in the center of the bottom of the capsule containing the embedding agent) and then baked at 60°C for 2 h to solidify into a block. The embedded block was cut into ultra-thin sections at 50–70 nm. After staining with uranium acetate and lead citrate, the sections were dried and observed under a transmission electron microscope (HT7700, Hitachi, Japan).

### Western Blotting

The method for western blotting detection was performed as described previously (Yu et al., 2013; Chen et al., 2015). Briefly, proteins were extracted from Schwann cells or sciatic nerve samples using Radioimmunoprecipitation assay (RIPA) buffer and then separated using sodium dodecyl sulfate polyacrylamide gel electrophoresis (SDS-PAGE) and transferred to a polyvinylidene fluoride (PVDF) membrane (microporous membrane). After blocking with 5% skim milk in Tris-buffered saline-Tween-20 (TBST) for 1 h at room temperature, the PVDF membrane was incubated with primary antibodies at 4°C overnight. After washing with TBST, the PVDF membrane was incubated with horseradish peroxidase (HRP)-conjugated secondary antibodies, and the immune complexes were visualized using Pierce^TM^ ECL Western Blotting substrate (Thermo Fisher Scientific, Waltham, MA, United States).

### Statistical Analysis

The statistical methods are shown in the figure legends. The statistical calculations were performed by using GraphPad Prism version 6.0 (GraphPad Software, Inc., La Jolla, CA, United States). All data are expressed in the form of the mean ± standard deviation (SD). The two-sided Student’s *t*-test was applied to determine statistical significance between groups. Ordinary one-way ANOVA test was used for comparison between more than two groups. *P* < 0.05 indicates statistical significance. In the figures, ^∗^*P* < 0.05, ^∗∗^*P* < 0.01.

## Results

### Differentially Expressed Injury-Related lncRNAs After Sciatic Nerve Injury

**FIGURE 1:**
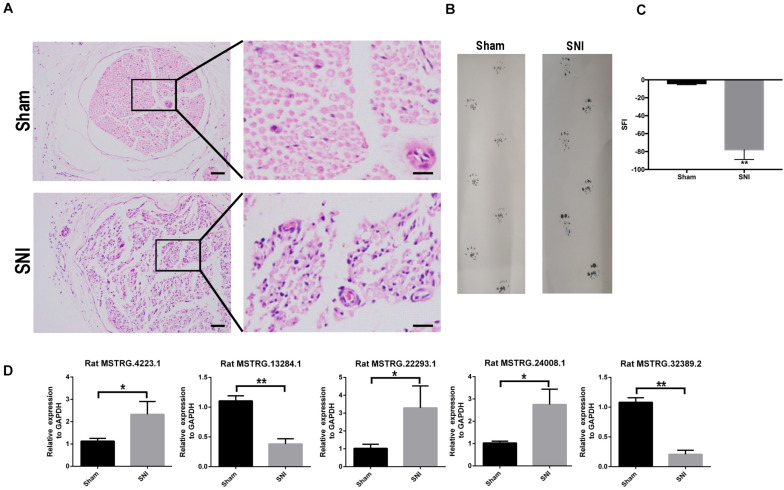
lncRNA expression increased after sciatic nerve injury. **(A)** Sciatic nerves, with or without injury, as analyzed using H&E staining. **(B)** Rat footprint function assessment (*n* = 6). **(C)** The SFI of **(B)** were measured using statistical analysis. Student’s *t*-test, **P < 0.01. Data are presented as the means ± SD. **(D)** qRT-PCR analysis showing the expression levels of injury-related lncRNAs after sciatic nerve injury that increased or decreased. Student’s *t*-test, *P < 0.05, **P < 0.01. Data are presented as the means ± SD. Scale bar: 50 and 10 μm in **(A)**. Data are representative of at least three independent experiments.

After SNI, the myelin sheath degenerated and the fibers became thinner and smaller. Cell apoptosis and the corresponding nerve tissue necrosis happened as the result (Figure 1A). After 2 weeks, the footprint function score in the SNI group was significantly lower than that in the normal group (*p* < 0.01) (Figures 1B,C), indicating that the SNI modeling was successful. Based on this model, RNA-seq was employed to screen for differentially expressed lncRNAs in the sciatic nerve tissues of the sham operation group and the SNI group. The results showed that compared with the sham operation group, 27 injury-related lncRNAs were upregulated and 13 injury-related lncRNAs were downregulated in the SNI group (Table 1 and Supplementary Figure 1). Based on the literature, five lncRNAs were finally screened for subsequent qRT-PCR verification (Rat lncRNAs *MSTRG.22293.1*, *MSTRG.24008.1*, *MSTRG.4223.1*, *MSTRG.13284.1*, and *MSTRG.32389.2*). The results showed that compared with those in the sham operation group, the expression levels of *MSTRG.22293.1*, *MSTRG.24008.1*, and *MSTRG.4223.1* were significantly increased and the expression levels of *MSTRG.13284.*1 and *MSTRG.32389.2* were significantly decreased (Figure 1D) in the SNI group, which was consistent with the RNA-seq results. Moreover, the expression of lncRNA *MSTRG.24008.1* reached the highest level on the sixth day (Supplementary Figure 2).

**TABLE 1:** Injury-related lncRNAs were screened by high-throughput sequencing.

Gene ID	Gene name	fc	Log2(fc)	*P*val	Gval	Regulation	Significant
MSTRG.13284	Itga8	−inf	−inf	≤0.001	0.00	Down	Yes
MSTRG.32389	Nsdhi	0.30	−1.72	≤0.001	0.00	Down	Yes
MSTRG.29881	Dclk3	0.28	−1.83	≤0.001	0.00	Down	Yes
MSTRG.15943	Sema5a	0.28	−1.84	≤0.001	0.00	Down	Yes
MSTRG.16216	Pex5i	0.27	−1.88	≤0.001	0.00	Down	Yes
MSTRG.15963	Car13	0.26	−1.96	≤0.001	0.00	Down	Yes
MSTRG.4407	Sgcd	0.25	−1.97	≤0.001	0.00	Down	Yes
MSTRG.1603	Svip	0.22	−2.18	≤0.001	0.00	Down	Yes
MSTRG.4419	RF00003	0.16	−2.63	≤0.001	0.00	Down	Yes
MSTRG.5120	AABR07030053.1	0.16	−2.67	≤0.001	0.00	Down	Yes
MSTRG.13154	Mtr	0.14	−2.81	≤0.001	0.00	Down	Yes
MSTRG.31298	RF00100	0.06	−4.11	≤0.001	0.00	Down	Yes
MSTRG.1559	AABR07003304.2	0.04	−4.78	≤0.001	0.00	Down	Yes
MSTRG.22293	Slc2a3	inf	inf	≤0.001	0.00	Up	Yes
MSTRG.24008	Kdm4a	inf	inf	≤0.001	0.00	Up	Yes
MSTRG.4223	AABR07029220.1	899.90	9.81	≤0.001	0.00	Up	Yes
MSTRG.6045	AABR07030791.1	311.43	8.28	≤0.001	0.00	Up	Yes
MSTRG.1004	Apoc4	247.58	7.95	≤0.001	0.00	Up	Yes
MSTRG.18534	Pax8	160.33	7.32	≤0.001	0.00	Up	Yes
MSTRG.21621	Polr1a	113.54	6.83	≤0.001	0.00	Up	Yes
MSTRG.8735	Tnr	56.41	5.82	≤0.001	0.00	Up	Yes
MSTRG.8274	Cntnap5c	53.39	5.74	≤0.001	0.00	Up	Yes
MSTRG.31011	Tns1	44.03	5.46	≤0.001	0.00	Up	Yes
MSTRG.18533	Pax8	43.98	5.46	≤0.001	0.00	Up	Yes
MSTRG.8181	Selplg	42.91	5.42	≤0.001	0.00	Up	Yes
MSTRG.31547	Cybb	23.53	4.56	≤0.001	0.00	Up	Yes
MSTRG.21260	Dqki	18.74	4.23	≤0.001	0.00	Up	Yes
MSTRG.2596	Itgad	15.95	4.00	≤0.001	0.00	Up	Yes
MSTRG.5500	Gngt2	9.22	3.20	≤0.001	0.00	Up	Yes
MSTRG.22632	Bhlhe41	7.75	2.95	≤0.001	0.00	Up	Yes
MSTRG.30416	Runx2	7.25	2.86	≤0.001	0.00	Up	Yes
MSTRG.7574	Fam20c	7.22	2.85	≤0.001	0.00	Up	Yes
MSTRG.30418	Runx2	6.66	2.74	≤0.001	0.00	Up	Yes
MSTRG.21310	Tbxas1	5.72	2.52	≤0.001	0.00	Up	Yes
MSTRG.22342	Cd4	5.03	2.33	≤0.001	0.00	Up	Yes
MSTRG.10221	Tns3	4.19	2.07	≤0.001	0.00	Up	Yes
MSTRG.30936	Plekhm3	4.10	2.94	≤0.001	0.00	Up	Yes
MSTRG.9233	Atf3	4.01	2.00	≤0.001	0.00	Up	Yes
MSTRG.23244	Tgfbr1	3.58	1.84	≤0.001	0.00	Up	Yes
MSTRG.19928	Sirpa	3.43	1.78	≤0.001	0.00	Up	Yes

### MSTRG.24008.1 Affects NLRP3 and MAL Expression via miR-331-3p

**FIGURE 2:**
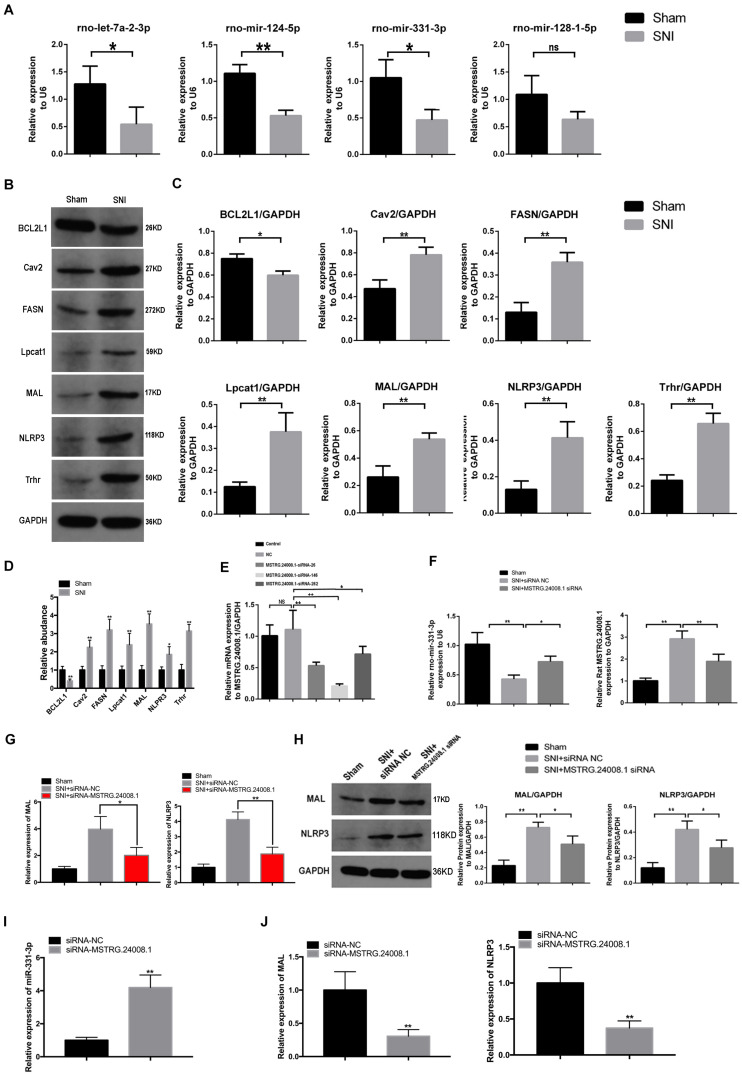
lncRNAs, miRNA, and relative protein levels changed after sciatic nerve injury. **(A)** qRT-PCR analysis showed decreased expression of miRNAs after sciatic nerve injury. Student’s *t*-test, *P < 0.05, **P < 0.01. Data are presented as the means ± SD. **(B)** Western blotting analysis of protein levels (BCL2L1, Cav2, FASN, Lpcat1, MAL, NLRP3, and Trhr) after sciatic nerve injury. **(C)** The relative protein levels on the western blots shown in **(B)** as measured using gray value analysis. Student’s *t*-test, *P < 0.05, **P < 0.01. Data are presented as the means ± SD. **(D)** qRT-PCR analysis showed the mRNA (BCL2L1, Cav2, FASN, Lpcat1, MAL, NLRP3, and Trhr) expression in the sciatic nerve tissue of the Sham and SNI groups. **(E)** qRT-PCR analysis showed the effects of different siRNAs on *MSTRG.24008.1* expression. One-Way ANOVA, *P < 0.05, **P < 0.01. Data are presented as the means ± SD. **(F)** qRT-PCR analysis showed the expression of miRNA-331-3p and *MSTRG.24008.1* were regulated by different siRNAs targeting on *MSTRG.24008.1.* Student’s *t*-test, *P < 0.05, **P < 0.01. Data are presented as the means ± SD. **(G)** The mRNA expression level of MAL and NLRP3 were affected by the siRNA targeting *MSTRG.24008.1* after sciatic nerve injury. **(H)** The protein levels of MAL and NLRP3 were affected by the siRNA targeting *MSTRG.24008.1* after sciatic nerve injury, as analyzed using western blotting. **(I)** The expression level of miR-331-3p was affected by the siRNA targeting *MSTRG.24008.1*. **(J)** The mRNA expression level of MAL and NLRP3 were affected by the siRNA targeting *MSTRG.24008.1*.

According to software prediction, four downstream miRNAs (let-7a-2-3p, miR-124-5p, miR-331-3p, and miR-128-1-5p) and six downstream proteins (caveolin 2 (Cav2), NLRP3, fatty acid synthase (FASN), lysophosphatidylcholine acyltransferase 1 (Lpcat1), thyrotropin releasing hormone receptor (Trhr), and MAL were selected for further verification. qRT-PCR was used to detect the expression of selected miRNAs. The results showed that compared with the sham operation group, the expression levels of let-7a-2-3p, miR-124-5p, and miR-331-3p were significantly reduced in the sciatic nerve tissues in the SNI group, while the expression of miR-128-1-5p showed a non-significant decreasing trend (Figure 2A). Western blotting detection of the six downstream proteins showed that compared with the sham operation group, the levels of Cav2, NLRP3, Fasn, Lpcat1, Trhr, and MAL in the sciatic nerve tissue in the SNI group were significantly increased and the level of BCL2 like 1 (BCL2L1) were significantly reduced (Figures 2B,C). In addition, the mRNA expression of Cav2, NLRP3, Fasn, Lpcat1, Trhr, and MAL in the sciatic nerve tissue in the SNI group and BCL2L1 were significantly reduced by qRT-PCR (Figure 2D). Therefore, based on the above results and software prediction, we finally hypothesized that *MSTRG.24008.1* affected the miR-331-3p/NLRP3/MAL signaling pathway to regulate the recovery of injured nerves.

To verify that *MSTRG.24008.1* affected the NLRP3 inflammasome and MAL protein levels through miR-331-3p, we designed an siRNA that could effectively downregulate *MSTRG.24008.1* expression (Figure 2E). qRT-PCR was used to detect the expression of related genes in the sciatic nerve after SNI. The results of qRT-PCR showed that, compared with that in the sham operation group, the expression of *MSTRG.24008.1* increased significantly and the expression of miR-331-3p decreased significantly in the SNI group 2 weeks after surgery. Compared with the SNI injury group, the expression of *MSTRG.24008.1* decreased significantly and the expression of miR-331-3p increased significantly in the siRNA interference group (Figure 2F). Moreover, the knockdown of *MSTRG.24008.1* significantly inhibited the mRNA expressions of MAL and NLRP3 (Figure 2G). Western blotting showed that compared with those in the sham operation group, the levels of MAL and NLRP3 increased significantly in the siRNA-NC group and the *MSTRG.24008.1* siRNA interference could significantly reverse the effect (Figure 2H). The expression of miR-331-3p increased significantly by the siRNA targeting *MSTRG.24008.1* treatment, which verified that *MSTRG.24008.1* affected the NLRP3 and MAL protein levels through miR-331-3p (Figure 2I). But the mRNA expression of MAL and NLRP3 decreased significantly after the siRNA targeting *MSTRG.24008.1* treatment (Figure 2J).

**FIGURE 3:**
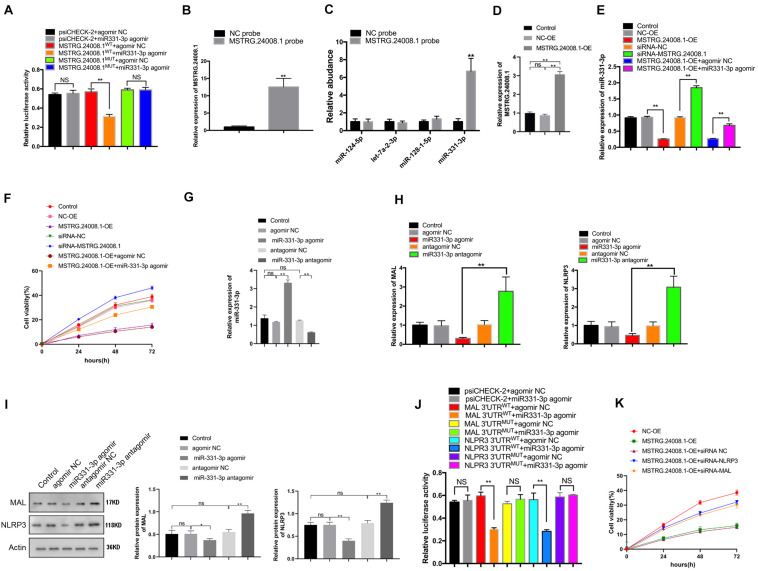
*MSTRG. 24008.1* regulated the expression of MAL and NLRP3 via miRNA-331-3p, thus regulating the proliferation of Schwann cells **(A)** Dual-luciferase reporter assay indicating that mir331-3p could bind with wild-type *MSTRG.24008.1*. Student’s *t*-test, **P < 0.01. Data are present as the means ± SD. **(B)** *MSTRG. 24008.1* in Schwann cells was pulled down by a *MSTRG. 24008.1*-specific probe and detected by qRT-PCR assay. **(C**) miRNA-331-3p was pulled down by using a *MSTRG. 24008.1*-specific probe. **(D)** qRT-PCR analysis demonstrated increased *MSTRG.24008.1* expression in Schwann cells upon *MSTRG.24008.1* overexpression. One-Way ANOVA, *P < 0.05, **P < 0.01. Data are presented as the means ± SD. **(E)** qRT-PCR analysis showed the regulatory effect of *MSTRG.24008.1* on miRNA-331-3p in Schwann cells. One-Way ANOVA, *P < 0.05, **P < 0.01. Data are presented as the means ± SD. **(F)** MTT assay was used to detect the proliferation curve of Schwann cells. **(G)** qRT-PCR analysis showing that the expression of miR-331-3p was altered after the transfection of the miR-331-3p agomir or antagomir in Schwann cells. One-Way ANOVA, **P < 0.01. Data are presented as the means ± SD. **(H)** The mRNA expression level of MAL and NLRP3 in response to miR-331-3p agomir or antagomir treatment as analyzed using qRT-PCR. **(I)** The protein levels of MAL and NLRP3 in response to miR-331-3p agomir or antagomir treatment as analyzed using western blotting. **(J)** Dual-luciferase reporter assay demonstrated that mir331-3p cold bind with MAL and NLPR3. Student’s *t*-test, *P < 0.05, **P < 0.01. Data are presented as the means ± SD. **(K)** The cell proliferation curve of Schwann cells treated with suppression of MAL or NRLP3.

The dual-luciferase reporter assay showed that the miR-331-3p agomir could significantly suppress the luciferase activity from the *MSTRG.24008.1*^WT^ construct, but had no effect on the luciferase activity from the *MSTRG.24008.1*^MUT^ construct (Figure 3A and Supplementary Figure 3). These results suggested that miR-331-3p bound directly to *MSTRG.24008.1*^WT^, but not to *MSTRG.24008.1*^MUT^. Then, the RNA pull-down assay was performed to evaluate whether *MSTRG.24008.1* could directly absorb these potential miRNAs. The biotinylated *MSTRG.24008.1* probe significantly pulled down *MSTRG.24008.1* upon *MSTRG.24008.1* overexpression (Figure 3B). Our results showed that miR-331-3p was the only one pulled down by MSTRG.24008.1 probe in SCs (Figure 3C). Overexpression of MSTRG.24008.1 was achieved successfully by transfection of the overexpression vector (Figure 3D). In addition, overexpression of *MSTRG.24008.1* dramatically downregulated, whereas *MSTRG.24008.1* siRNA upregulated, the expression of miR-331-3p in Schwann cells. The miR-331-3p agomir could reverse the effect caused by overexpression of *MSTRG.24008.1* (Figure 3E). Importantly, the MTT assay results showed that siRNA-mediated downregulation of *MSTRG.24008.1* promoted Schwann cell proliferation, whereas overexpression of *MSTRG.24008.1* inhibited Schwann cell proliferation, and this effect could be reversed by miR-331-3p agomir (Figure 3F). The results of Edu were consistent with those of MTT (Supplementary Figures 4A,B). Thus, these results suggested that *MSTRG.24008.1* negatively regulated miR-331-3p expression to inhibit Schwann cell proliferation.

We next further explored the role of the regulation of MAL and NLRP3 targeted by miR-331-3p in repair of peripheral nerve injury. qRT-PCR showed that the expression of miR-331-3p was significantly increased in Schwann cells transfected with the miR-331-3p agonist and significantly reduced in cells transfected with the miR-331-3p antagomir (Figure 3G). Overexpression of miR-331-3p inhibited mRNA expression of MAL and NLRP3 (Figure 3H). Western blotting showed that the miR-331-3p agomir notably downregulated, and the miR-331-3p antagomir upregulated the levels of NLRP3 and MAL (Figure 3I). Dual-luciferase reporter assay showed that the miR-331-3p agomir significantly reduced the luciferase activity from NLRP3^WT^ and MAL^*WT*^, but did not affect the luciferase activity from NLRP3^MUT^ and MAL^MUT^ (Figure 3J). Then we designed siRNA that could effectively down-regulate NLRP3 and MAL expression for subsequent experiments. Importantly, the MTT results showed that overexpression of *MSTRG.24008.1* could inhibit Schwann cells proliferation, which was reversed by adding siRNA-NLRP3 or siRNA-MAL (Figure 3K). The results of Edu were also consistent with those of MTT (Supplementary Figures 4C,D). These outcomes indicated that NLRP3 and MAL were downstream targets of miR-331-3p and MSTRG.24008.1. Therefore, interfering with *MSTRG.24008.1* expression could increase the levels of miR-331-3p, thereby decreasing NLRP3 and MAL levels to promote Schwann cell proliferation. As IL-1beta and IL-18 are the downstream targets of NLRP3, the expression of IL-1beta and IL-18 were also detected. The mRNA expression of IL-1beta and IL-18 decreased significantly by the siRNA targeting *MSTRG.24008.1* treatment (Supplementary Figure 5).

### Effects of MSTRG.24008.1 on Schwann Cells and Neurons

**FIGURE 4:**
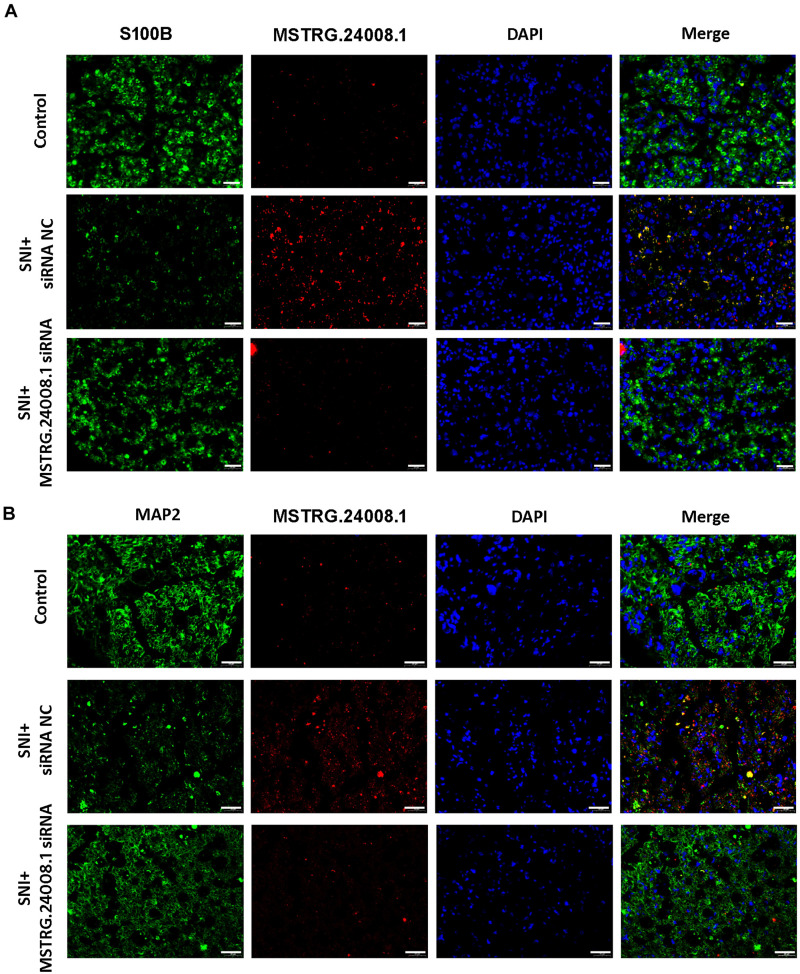
The expression levels of S100B and MAP2 detected in SNI using fluorescence *in situ* hybridization. **(A)** Fluorescence *in situ* hybridization indicated that the expression of *MSTRG.24008.*1 was significantly increased and that of S100B was significantly decreased in the SNI group compared with those in the Sham group; however, the expression of *MSTRG.24008.1* was significantly decreased and that of S100B was significantly increased in the SNI group treated with the *MSTRG.24008.1* siRNA compared with those in the SNI group. **(B)** Fluorescence *in situ* hybridization indicated that the expression of *MSTRG.24008.1* was increased, and that of MAP2 was significantly decreased in the SNI group compared with those in the Sham group; however, the expression of *MSTRG.24008.1* was significantly decreased and that of MAP2 was significantly increased in the SNI group treated with the *MSTRG.24008.1* siRNA compared with those in the SNI group. Scale bar: 20 μm **(A,B)**.

To further investigate the effects of *MSTRG.24008.1* on Schwann cells and neurons, we performed FISH analysis. S100 calcium binding protein B (S100B) is a common immunohistochemical marker of Schwann cells after peripheral nerve injury, which is expressed mostly in Schwann cells in the PNS, regardless of the Schwann cells phenotype (Tomlinson et al., 2020). Microtubule-associated protein 2 (MAP2) is a neuron-specific protein that binds to microtubules (Ferralli et al., 1994), which is found abundantly in neural cells. Studies found that reduction in MAP2 immunoreactivity was a sensitive and quantifiable early marker of neural injury in rats (Lingwood et al., 2008). Therefore, we used S100B and MAP2 as markers for Schwann cell proliferation and neuronal cells damage, respectively. The FISH results (Figures 4A,B) showed that, in the damaged sciatic nerve tissue, the level of *MSTRG.24008.1* was significantly increased and the expression of S100B was significantly decreased, whereas the level of MAP2 was also reduced significantly at 2 weeks after surgery compared with those in the Sham group. The co-localization of *MSTRG.24008.1* and S100B indicated that *MSTRG.24008.1* expressed in the Schwann cell cytoplasm. Also, the co-localization of *MSTRG.24008.1* and MAP2 demonstrated that *MSTRG.24008.1* expressed in the neuronal cytoplasm. After Knockdown of *MSTRG.24008.1*, the expression of *MSTRG.24008.1* was significantly reduced; However, S100B and MAP2 expression were significantly increased in Schwann cells and neurons respectively. Interference with *MSTRG.24008.1* expression could promote neuron repair and Schwann cell reproduction, thereby better repairing the damage sciatic nerve tissue.

### MSTRG.24008.1 Promotes the Regeneration of Injured Sciatic Nerve *in vivo*

**FIGURE 5:**
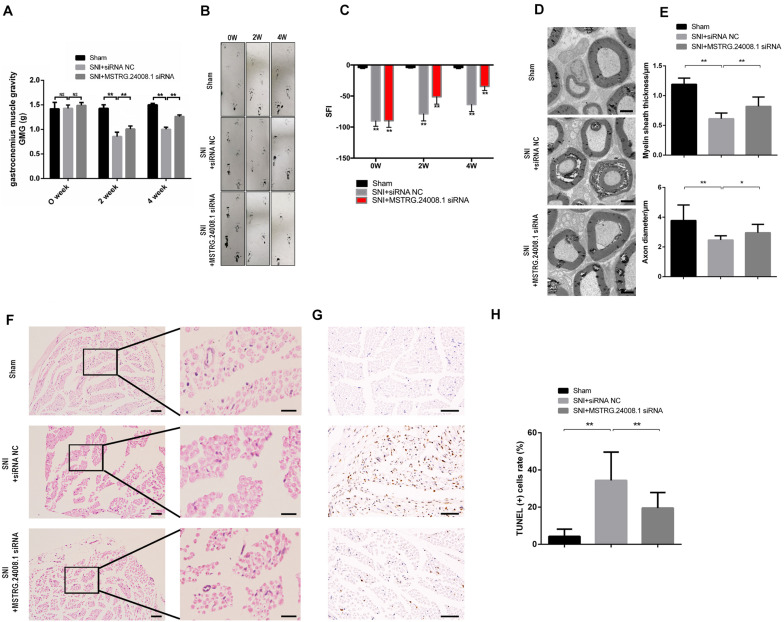
*MSTRG.24008.1* regulates the regeneration of SNI *in vivo*. **(A)** GMG in the SNI + MSTRG.24008.1 siRNA group increased significantly. Student’s *t*-test, **P < 0.01. Data are represented as the means ± SD. **(B)** Rat footprint function assessment (*n* = 6). **(C)** The SFI from **(B)** were measured using statistical analysis. Student’s *t*-test, **P < 0.01. Data are represented the means ± SD. **(D)** TEM showing that the myelin sheath thickness and axon diameter of SNI increased after *MSTRG.24008.1* siRNA treatment (*n* = 6). **(E)** The myelin sheath thickness and axon diameter in **(D)** measured using Image-Pro Plus 6.0 software. Student’s *t*-test, *P < 0.05, **P < 0.01. Data are represented as the means ± SD. **(F)** Sciatic nerves analyzed using H&E staining. **(G)** Sciatic nerves analyzed using TUNEL staining. **(H)** The number of apoptotic cells in **(G)** were counted. Student’s *t*-test, *P < 0.05, **P < 0.01. Data are represented as the means ± SD. Scale bar: 5 μm in **(D)**, 50 and 10 μm in **(F)**, and 20 μm in **(G)**. Data are representative of at least three independent experiments.

After establishing rat sciatic nerve injury model, the recovery of sciatic nerve was evaluated by gastrocnemius muscle gravity (GMG), footprinting function and score analysis. The ultrastructures of the regenerating nerve, myelin sheath regeneration, and apoptosis of the injured sciatic nerve were observed using electron microscopy, H&E and TUNEL staining, to verify the repairing effects of the *MSTRG.24008.1* siRNA on the injured sciatic nerve. The results of gastrocnemius wet weight measurement showed that there was no significant difference in GMG between the sham operation group, the SNI + siRNA NC group, and the SNI + MSTRG.24008.1 siRNA group at 0 weeks after surgery. Compared with that of the sham operation group, the GMG in the rats of the SNI + siRNA NC group was significantly reduced at 2 and 4 weeks after surgery. Compared with that of the SNI + siRNA NC injury group, the GMG in the rats in the SNI + MSTRG. 24008.1 siRNA group increased significantly (Figure 5A), which showed that knockdown of *MSTRG.24008.1* expression could increase the GMG in the SNI model. The footprint function and scoring analysis results showed that compared with those in the sham operation group, the rats in the SNI + siRNA NC group were injured notably at 0 weeks after surgery, and there was no significant difference in the degree of injury between the SNI + MSTRG. 24008.1 siRNA group and the SNI + siRNA NC group. At 2 and 4 weeks after surgery, compared with those in the sham operation group, the rats in the SNI + siRNA NC group were still significantly injured. However, compared with the rats in the SNI + siRNA NC injury group, the rats in the SNI + MSTRG. 24008.1 siRNA group had less injury (Figures 5B,C). Thus, interference with *MSTRG.24008.1* expression could reduce the degree of injury in the SNI model.

The electron microscopy results (Figure 5D) showed that, in the sham operation group, the morphology and structure of rat sciatic nerve were normal. The axon was not shrunken and swollen, and the subcellular structure was distributed normally. However, in the SNI + siRNA NC group, the structure of myelin sheath was loose and distorted, and vacuole-like degeneration appeared, with an enlarged lamellar space and axon atrophy. In the SNI + MSTRG.24008.1 siRNA group, the myelin sheath tissue tended to be intact, and the vacuole-like degeneration decreased. The structure was relatively neat and complete, and there was no obvious axonal atrophy. The myelin thickness and axon diameter of each group were measured. Compared with that in the sham operation group, the myelin thickness of the SNI + siRNA NC group was significantly reduced (*p* < 0.01) and the axon diameter also decreased significantly (*p* < 0.01). Compared with that in the SNI + siRNA NC group, the myelin thickness of the SNI + MSTRG.24008.1 siRNA group increased significantly (*p* < 0.01), and the axon diameter also increased (*p* < 0.05) (Figure 5E).

The HE staining results showed that, in the sham operation group, the anatomy of the nerves was normal (Figure 5F). In the SNI + siRNA NC group, the nerve tissue was loosely arranged, the myelin sheath was damaged, and the number of myelin fibers was reduced. In the SNI + MSTRG.24008.1 siRNA group, the degree of neural tissue damage was intermediate between that of the sham operation group and the SNI + siRNA NC group.

The TUNEL results showed (Figure 5G) that, compared with the sham operation group, the apoptosis rate of sciatic nerve cells in the SNI group increased significantly increased. Compared with the SNI + siRNA NC group, the apoptosis rate in the SNI + *MSTRG.24008.1* siRNA group was significantly reduced (*p* < 0.01) (Figure 5H). The results suggested that knockdown of *MSTRG.24008.1* expression inhibited the apoptosis of sciatic nerve cells caused by SNI. The above results verified that knockdown of *MSTRG.24008.1* expression promoted the regeneration of injured sciatic nerves *in vivo*.

## Discussion

SCs, a subset of peripheral glial cells, are 70–80% of the components of the peripheral nervous system (Madduri and Gander, 2010). They play an important role in the regeneration of nerves and the recovery of motor function after the peripheral nerve injury. Several studies have concluded that SCs promote nerve regeneration and the recovery of motor function by secreting a variety of neurotrophic factors, clearing damaged axons and myelin, and providing structural guidance (Liu et al., 2013; Zhang and Rosen, 2018). For this reason, SCs have been a frequent target in strategies to promote repair of peripheral nerve injury. LncRNAs are involved in the damage repair process of a variety of cells (Liang and Hu, 2019; Li et al., 2019; Wang et al., 2019), and have become a research hotspot. Axon outgrowth and Schwann cell proliferation are two of the major processes of neuroregeneration (Grinsell and Keating, 2014; Liu et al., 2019). Emerging studies concerning lncRNA regulation of neuroregeneration mostly revealed that the lncRNAs participate in the two mechanisms mentioned above. For example, the knockdown of lncRNA *Silc1* promote neural outgrowth (Perry et al., 2018). LncRNAs *BC089918* and *uc.217* were downregulated after SNI, and the silencing of lncRNAs *BC089918* and *uc.217* could promote neurite outgrowth (Yao et al., 2015). The roles of lncRNAs in Schwann cells have also been gradually revealed (Yao and Yu, 2019). For instance, lncRNA *NON-MMUG014387* promotes the proliferation of Schwann cells surrounding lesions by targeting collagen triple helix repeat c containing 1 (Cthrc1) and activating the Wnt/PCP signaling (Pan et al., 2017). LncRNA *TNXA-PS1* expression was decreased after sciatic nerve injury, and its downregulation could promote Schwann cell migration (Yao et al., 2018). LncRNA *NEAT1* could promote the proliferation and migration of Schwann cells and axon outgrowth by regulating the miR-34a/SATB homeobox 1 (Satb1) axis (Liu et al., 2019). However, most of these studies only included one of the mechanisms of neuroregeneration and lacked *in vivo* experiments for further verification. Therefore, we performed an integrated experiment from molecules to rats to verify our proposed mechanism. We also found that MSTRG.24008.1 affected neuroregeneration by regulating both neuron repair and Schwann cell proliferation.

In this study, we first found that a series of injury-related lncRNAs, miRNAs, and proteins were differentially expressed in the rat sciatic nerve after SNI. We subsequent chose lncRNA *MSTRG.24008.1*, one of significantly upregulated lncRNAs after SNI, as our research target. Using bioinformatic analysis, we found that *MSTRG.24008.1* might act as a competitive endogenous RNA (ceRNA) for downstream miRNA miR-331-3p to regulate sciatic nerve neuroregeneration after SNI.

MicroRNA miR-331-3p is a recognized tumor suppressor that impedes tumor cell proliferation and migration in various types of cancer (Liu et al., 2014; Du et al., 2020; Peng et al., 2020). In the CNS, miR-331-3p can inhibit the inflammatory response by targeting the downstream protein NLR family pyrin domain containing 6 (NLRP6) (Nie et al., 2020). However, the roles of miR-331-3p in the PNS were unclear. We found that miR-331-3p expression was downregulated after SNI. Further experiments *in vitro* demonstrated that the miR-331-3p agomir could promote Schwann cell proliferation, subsequently promoting neuroregeneration. Using bioinformatic analysis, we found that NLRP3 and MAL were two downstream target proteins of miR-331-3p. NLRP3 is one type of inflammasomes. Inflammasomes, cytoplasmic polymer protein complexes in essence, are formed by the detection of pathogen-associated molecular patterns (PAMPs) or danger-associated molecular patterns (DAMPs) by host cells. After NLRP3 inflammasome formation, procaspase-1 is activated, then cleave and activate the precursors of the cytokines interleukin (IL)-1β and IL-18, leading to an inflammatory response (Cui et al., 2020). The NLRP3 inflammasome has been verified to be involved in neurodegenerative and CNS diseases (Li et al., 2020; Zhang et al., 2020; Ismael et al., 2021). However, its role in the PNS remained controversial. A study showed that the expression levels of NLRP3 and downstream IL-1β were increased after SNI, which led to inflammatory responses, thereby impeding the neural recovery. In addition, knockout of *NLRP3* could promote neural recovery (Pirzada et al., 2020). Intriguingly, another study demonstrated that the NLRP3 inflammasome did not play a role in the functional recovery after peripheral nerve injury (Ydens et al., 2015). Moreover, some studies claimed that IL-1β could promote functional recovery, Schwann cell proliferation, and neurite outgrowth (Temporin et al., 2008; Nadeau et al., 2011). Emerging studies indicated that MAL was implicated in tumor promotion and suppression (Lara-Lemus, 2019). Importantly, overexpression of MAL could impede myelination in the PNS, which was consistent with our results (Buser et al., 2009; Schmid et al., 2014). In the present study, we first found that *MSTRG.24008.1* was differentially expressed in the SNI model. Following bioinformatic analysis, we performed further experiments *in vitro*. After overexpression of *MSTRG.24008.1* in Schwann cells, we found that miR-331-3p expression was decreased. In addition, the levels of the downstream factors NLRP3 and MAL increased, which impeded neural recovery. Dual-luciferase reporter assays and siRNA interference experiments were performed to verify our hypothesis. Importantly, both siRNA-NLRP3 and siRNA-MAL could promote Schwann cell proliferation. Combined with further *in vivo* experiments, our results supported the view that downstream proteins NLRP3 and MAL negatively affected neuroregeneration. However, we did not detect the expression of interleukin 1 beta (IL-1β), which was a limitation of our study. Furthermore unraveling these controversies and determining the downstream signaling pathway of NLRP3 and MAL that affects neuroregeneration require further experiments.

## Conclusion

The results of the present study showed that lncRNA *MSTRG.24008.1* expression increased after sciatic nerve injury. Interference with *MSTRG.24008.1* expression in rats could downregulate the expressions of NLRP3 and MAL by increasing miR-331-3p expression, which in turn promoted Schwann cell proliferation and neuron repair, thus promoting neural recovery.

## Data Availability Statement

The datasets presented in this study can be found in online repositories. The names of the repository/repositories and accession number can be found below: (CRA003832).

## Ethics Statement

The animal study was reviewed and approved by the Research Ethics Committee of Shanghai General Hospital.

## Author Contributions

GY, YP, and HL conceived and designed the studies. GY, YP, ZL, PW, and RW performed most of the experiments and analyzed the data. YL and ZL conducted some of the animal studies and cell experiments. GY and HL provided essential reagents and assisted with the experimental design and data analysis. GY and YP wrote the manuscript. All authors contributed to the article and approved the submitted version.

## Conflict of Interest

The authors declare that the research was conducted in the absence of any commercial or financial relationships that could be construed as a potential conflict of interest.
