# Supplementary material for: Long Non-coding RNA MSTRG.24008.1 Regulates the Regeneration of the Sciatic Nerve via the miR-331-3p–NLRP3/MAL Axis
Source: Front Cell Dev Biol. 2021 Jun 4;9:641603. doi: 10.3389/fcell.2021.641603 (PMC8213216; doi:10.3389/fcell.2021.641603)

Supplementary Figure 1


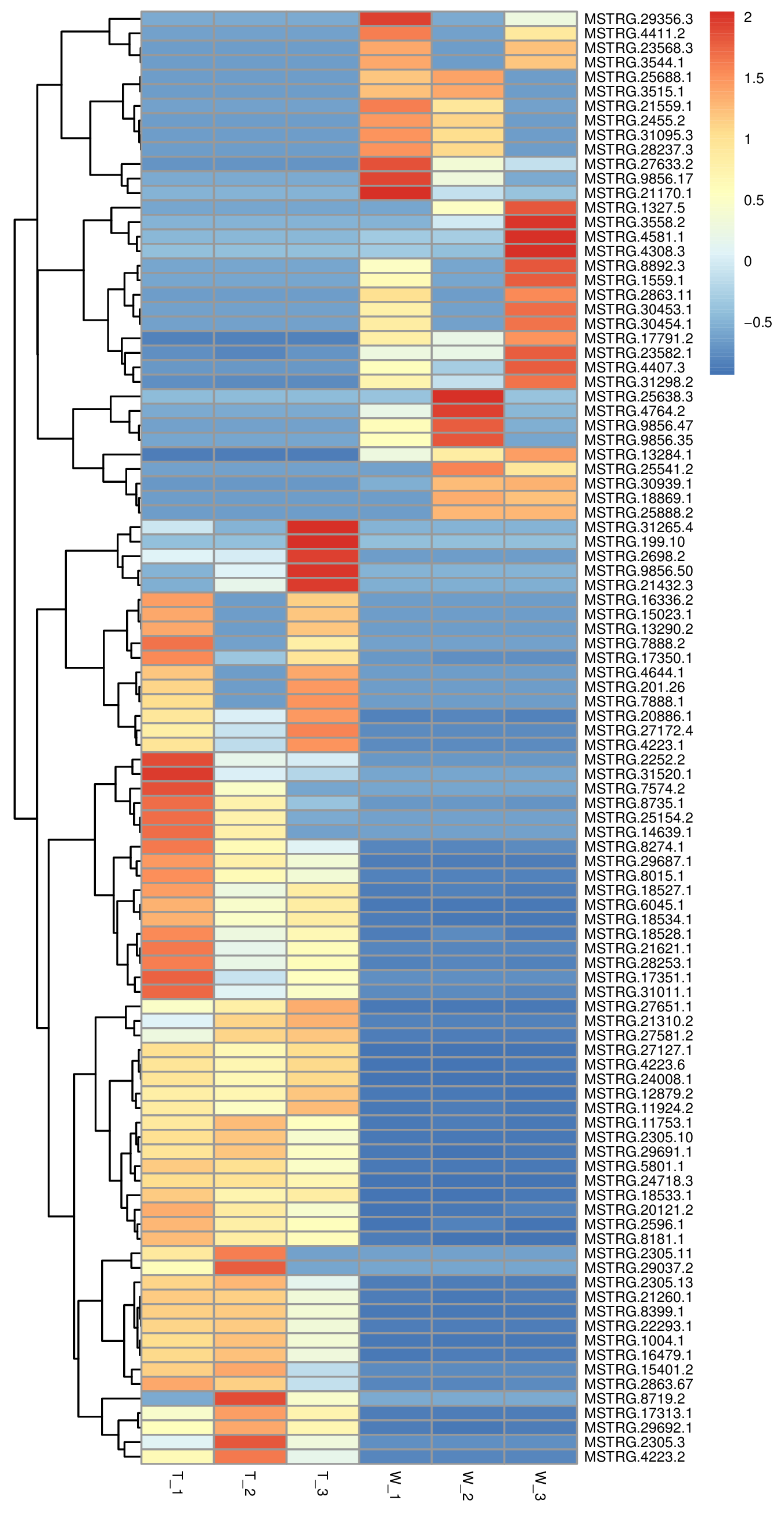


Supplementary Figure 2


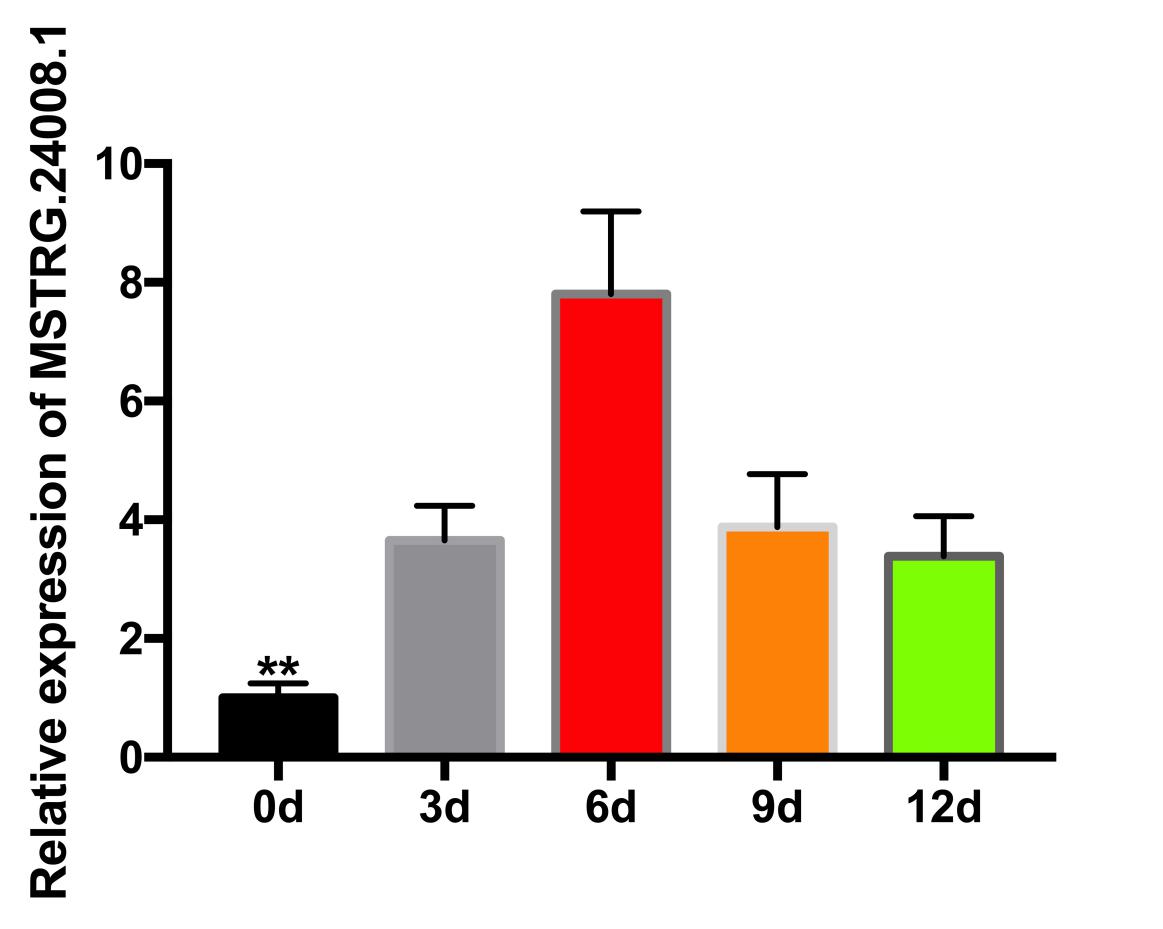


Supplementary Figure 3


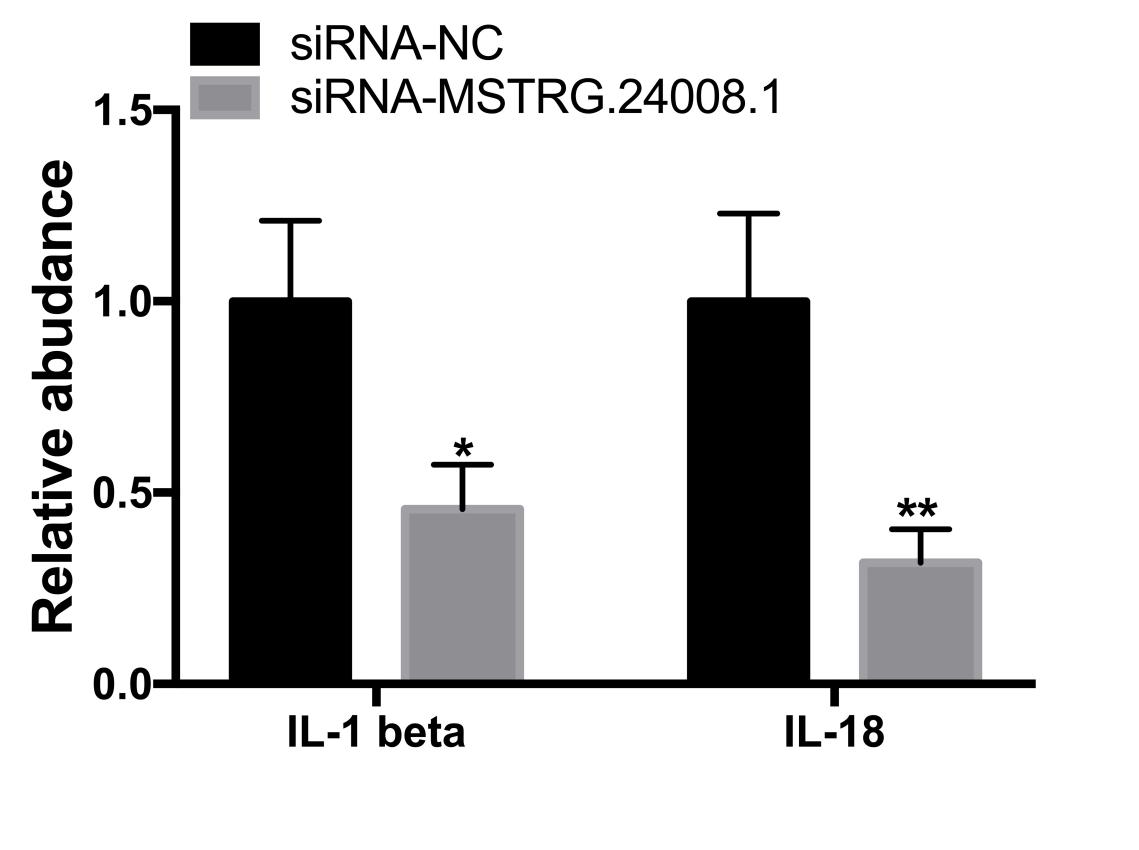


Supplementary Figure 4


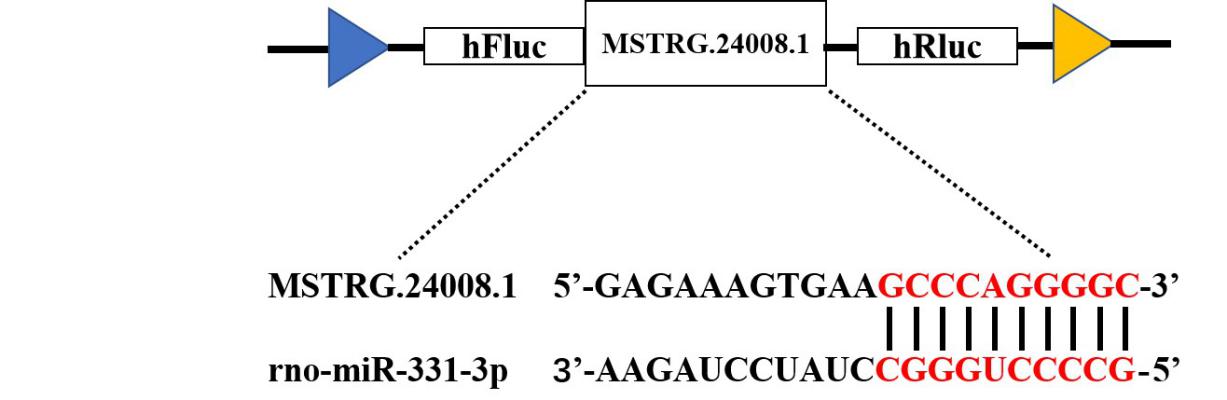


Supplementary Figure 5


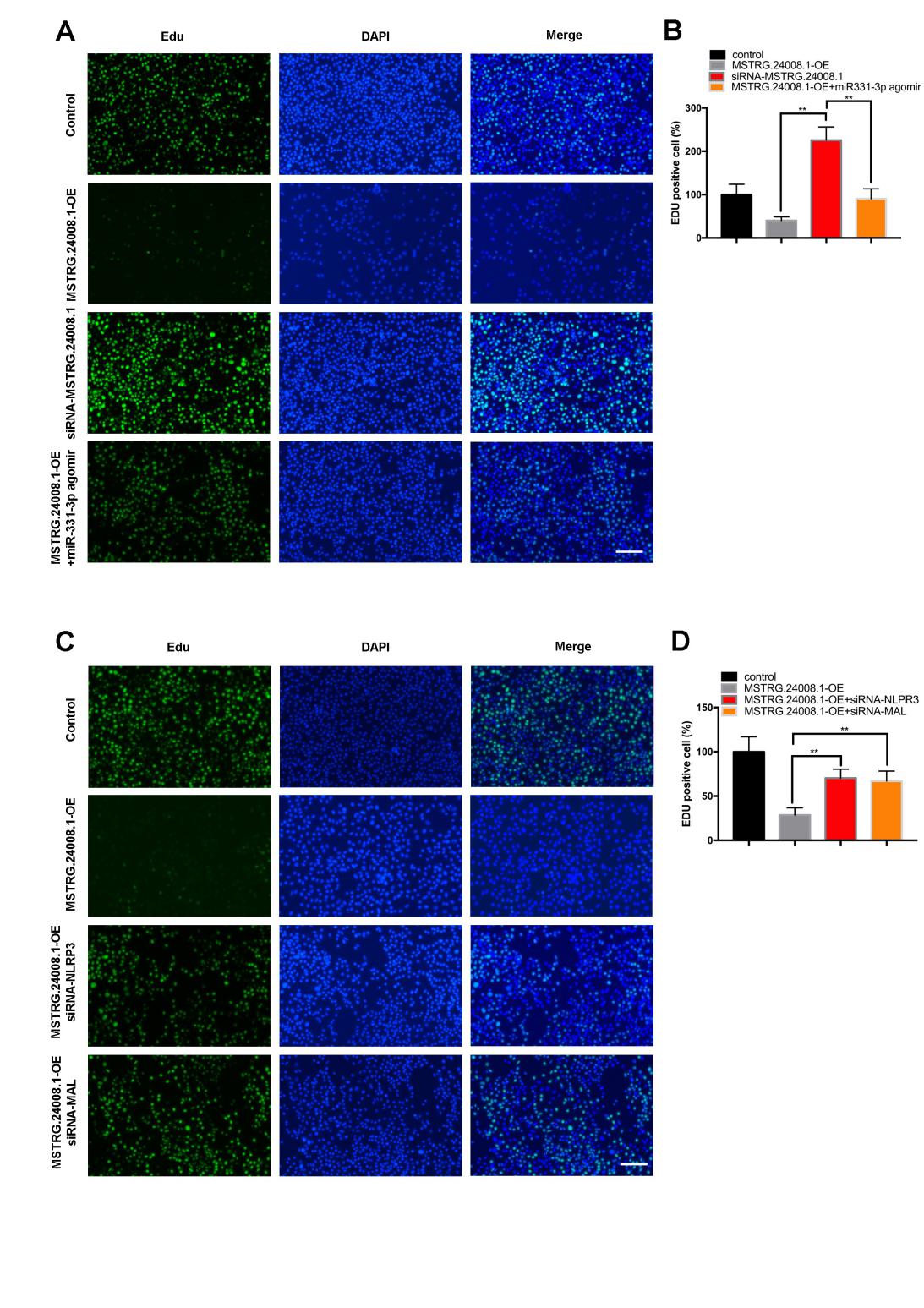

Supplement: Supplementary file 1 [file Table_1.DOCX]
